# Level of technical efficiency and associated factors among health centers in East Gojjam Zone, Northwest Ethiopia: an application of the data envelopment analysis

**DOI:** 10.1186/s12913-024-10843-1

**Published:** 2024-03-21

**Authors:** Anteneh Lamesgen, Belayneh Mengist, Elyas Melaku Mazengia, Bekalu Endalew

**Affiliations:** 1 Department of Public Health, College of Health Sciences, Debre Markos University, Debre Markos, Ethiopia; 2 The Institute for Mental and Physical Health and Clinical Translation (IMPACT), School of Medicine, Deakin University, Victoria, Australia

**Keywords:** Technical efficiency, Data envelopment analysis, Health center, Tobit regression

## Abstract

**Background:**

Besides the scarcity of resources, inefficient utilization of available health service resources has been the bottleneck to deliver quality health services in Ethiopia. However, Information regarding the efficiency of health service providers is limited in the country. Health service managers and policy makers must be well informed about the efficiency of health service providers and ways of using limited resources efficiently to make evidence-based decisions. This study aimed to assess the level of technical efficiency and associated factors among health centers in East Gojjam Zone, Northwest Ethiopia.

**Methods:**

A facility-based cross-sectional study was conducted among 27 randomly selected health centers in East Gojjam zone, Northwest Ethiopia, from October 30, 2022, to April 30, 2023. Using an interviewer-administered questionnaire and document review checklist, health centers’ data was collected and entered to Epi-Data version 4.6. The data was exported to Microsoft office excel and Stata version 14 for analysis. A two-stage output-oriented data envelopment analysis with a variable return to scale assumption was employed to determine the level of technical efficiencies. Finally, the tobit regression model was applied to identify the associated factors at 5% level of significance.

**Results:**

In this study, 59.3% of the health centers were technically efficient. The mean technical efficiency score of the health centers was 0.899 ± 0.156. Inefficient health centers could provide more 22, 433 outpatient visits, 1,351 family planning visits, 155 referral services, 206 skilled deliveries and 385 fully vaccinations of children if they were technically efficient as their peer health centers for the same year. From the tobit regression, the catchment population and number of administrative staffs were statistically significant determinants of the technical efficiency of health centers.

**Conclusions:**

The mean technical efficiency of the health centers in East Gojjam zone, Northwest Ethiopia was high. However, nearly half of the health centers were technically inefficient, which indicates the exitance of a space for further improvements in the productivity of these health centers. Employing excess number administrative staffs (above the optimal level) should be discouraged and selecting appropriate sites where the health centers to be constructed (to have large catchment population coverage) could improve the productivity of health centers.

**Supplementary Information:**

The online version contains supplementary material available at 10.1186/s12913-024-10843-1.

## Background

Efficient use of resources has become the fundamental strategy of policy makers within most country’s health systems in the world. Such explosion of interest in measuring efficiency in health systems is attributed to intensified concerns with the costs of health care, increased demands for public accountability, and improved capabilities for measuring performances [1].

Three main categories of efficiency measurements are known in health care. Namely, allocative efficiency, technical efficiency, and overall economic efficiency [2]. Allocative efficiency (AE) is used to scrutinize whether limited resources are directed towards producing the correct mix of outputs or the entity under examination uses an optimal mix of inputs to produce its chosen outputs. Technical efficiency (TE) indicates the extent to which a given decision making unit (DMU) is minimizing the use of inputs in producing its chosen outputs or maximizing its outputs given its chosen level of inputs. If a DMU uses its resources in technical and allocative efficient way, then it can be said to have achieved its total economic efficiency [3]. Since technical efficient is the primary footstep to attain the level of allocative efficiency and even the overall economic efficiency for a given DMU, we assessed the TE of health centers by considering the health center as a single DMU.

Health centers are health facilities which provides promotive, preventive, curative and rehabilitative outpatient care including basic laboratory and pharmacy services with the capacity of 10 beds for emergency and delivery services in the primary health care system of Ethiopia [4].

The Ethiopian primary health care system ends at the primary hospitals at the top which provides primary curative, preventive and rehabilitative services with referral from health centers or directly. Next to the primary hospitals are health centers. These units are supposed to provide service for an average population of 25,000. Basic curative, preventive and rehabilitative services are delivered in the health centers. The nearest service point to the community are the health posts. Health posts provide mostly preventive and promotive services as well as some basic curative care home to home, outreach and at facility. There is a referral and administrative linkage between these three entities. Health center is a referral point for health posts. Similarly, primary hospitals are referral centers for health centers [5].

The health sector of Ethiopia is facing with scarcity of resources. It is one of the underfinanced sectors in the country; the share of government health expenditure accounted 1.4% of the country’s GDP in 2016/17, which is lower than 1.9% in low-income countries for the same year, and well below the global average of 5.3% [6]. Together with this constraint, inefficient use of available resources can be taken as a double burden for the country to deliver quality health services for its citizens.

There are studies on the assessment of health facilities’ efficiency globally [7–11] and in different countries of Africa [12–14]. However, such evidence is limited in Ethiopia. Few articles [15–20] are documented on the technical efficiency of health facilities in some areas of the country. Getachew [20] observed the technical efficiency of selected hospitals in Ethiopia. Ali, et al. [15] assessed the technical efficiency of selected hospitals in eastern Ethiopia. The study conducted in Tigray region [16] assessed the performance of health posts. The technical efficiency of 12 public hospitals was assessed by the study conducted in Northwest Ethiopia [18]. The other study conducted in Southwest Ethiopia [19] assessed the technical efficiency of health posts and health centers. Each of these studies were based on an input-oriented data envelopment analysis, which is mainly focus on an input minimization for a given set of outputs in a production process [1]. However, there is an evenly resource allocation for similar health facilities in Ethiopia [21] and the input side could not merely evaluate their efficiency levels, rather the quantity of outputs they produce from their services can be used to estimate their performances. Besides this, studies on the lower-level health facilities in the Northwest region of Ethiopia, where quality of health care could be compromised due to the scarcity and/or inefficient use of resources, are too minimal. To this end, we were interested to evaluate the technical efficiency of health centers in East Gojjam zone, Northwest Ethiopia using the output-oriented data envelopment analysis. We have also observed the effect of organizational and environmental related factors of health facilities, which are discussed on literatures, on the technical efficiency of health centers.

## Methods

### Study design and setting

A facility-based cross-sectional study was conducted from October 30, 2022 to April 30, 2023. The study was conducted among health centers located in East Gojjam Zone, Northwest Ethiopia. The Ethiopian health care system is structured into a three-tier health system; the primary, secondary, and tertiary levels of health care. The primary level of health care is composed of primary hospital, health center, and health post [22]. The East Gojjam Zone, where the study was conducted has 18 health districts. In this Zone, there are 10 public hospitals, 101 health centers, and 430 health posts [23].

### Study participants

The source population of this study was all health centers located in East Gojjam Zone, Northwest Ethiopia and the study population was all health centers located in randomly selected districts of the East Gojjam Zone. Health centers which were functional (provide health services) for the year (July 2021 to June 2022) were included in this study.

### Sample size calculation and sampling procedure

We used the WHO tool for assessing operationality of district health systems [24] as guideline to determine the sample size. Accordingly, nine health districts in East Gojjam Zone were selected randomly. Using simple random sampling technique three health centers were selected from each health districts to have a total of 27 sample health centers. The rule of thumb recommended in [25, 26], the number of DMUs should be at least twice the sum of inputs and outputs, was used to determine the number of inputs and outputs for the efficiency analysis. Accordingly, four inputs and five outputs were included.

### Study variables

The technical efficiency score of the health centers was the dependent variable. Inputs and outputs used to compute the technical efficiency were identified after reviewing related studies [18, 19]. The inputs were number of administrative staffs, number of clinical staffs, number of beds, and expenditures for recurrent materials including pharmaceuticals, water, electricity, fuel, and maintenances. The outputs were the number of outpatient visits, number of referrals (referral out), number of skilled deliveries, number of family planning visits, and number of fully vaccinated children. The independent variables were composed of organizational and environmental characteristics of health centers. Organizational characteristics include; service year of the health center, educational status of the head, managerial service year of the head, and patient waiting time. Environmental characteristics include; catchment population, availability of nearby health facility, and location of the health center.

### Data collection procedure

Data was collected using the questionnaire which was composed from interviewer-administered questions and document review checklist (Additional file 1). This questionnaire was prepared after reviewing related studies [18, 19] and adapting the WHO tool for assessing the operationality of district health systems [24]. Environmental and organizational characteristics of health centers were accessed through an interviewer-administered questionnaire from the heads of each health center. All input and output data of health centers for one Ethiopian fiscal year (July 2021 to June 2022) were collected by reviewing documents using the checklist. The input and output data were collected from the health centers’ head, plan, human resource, and finance offices. For the input data, a bottom-up costing approach which considers the cost of each item and multiplies with the number of items to get the total cost [27] from the provider perspective was employed. Finally, all information collected in Ethiopian Birr (ETB) was changed into United States dollar (US$) using the June 2022 exchange rate [28].

### Data quality assurance

Data collectors who had a BSc degree in health science fields were recruited, and training was given for them before the data collection. The data collection tool was first prepared in English, then it was translated into Amharic, and it was then back translated into English to check its consistency. A pretest of the tool was done on three health centers from the source population and a revision was made on it to eliminate misunderstandings. Beside this, the tool was exposed for judgements of health economics experts and amendments was done after taking necessary comments. During the data collection period, the tool was checked every day for completeness and feedback was given to the data collectors whenever they faced challenges.

### Data processing and analysis

After the data was collected, it was checked for completeness, cleaned, entered into Epi-Data version 4.6, and exported to Microsoft office excel to set the total summarized inputs and outputs of each health centers and then to STATA version 14 for the statistical analysis. The technical efficiency for each health centers was computed using DEA Program version 2.1 (DEAP 2.1) developed by Tim Coelli [29]. The estimated efficiency scores were regressed against organizational and environmental characters of the health centers using the Tobit regression model. Finally, significant factors were identified at 5% level of significance.

### Measure of technical efficiency

Data Envelopment Analysis (DEA) is a non-parametric linear programming technique that develops an efficiency frontier based on observed facts to calculate a given organization’s efficiency relative to other organizations’ performance producing the same good or service. It is a data-oriented approach, for evaluating the performance of a set of peer entities called Decision-Making Units (DMUs), which convert multiple inputs into multiple outputs. As stated in [1, 3, 30, 31], technical efficiency is defined as the ratio of the weighted sum of outputs of a given DMU divided by the weighted sum of its inputs, which is given in Eq. 1.

1 $$\mathrm{Technical}\;\mathrm{efficiency}=\frac{\mathrm{Weighted}\;\mathrm{sum}\;\mathrm{of}\;\mathrm{outputs}}{\mathrm{Weighted}\;\mathrm{sum}\;\mathrm{of}\;\mathrm{inputs}}$$

In the DEA of technical efficiency, if the emphasis is on reducing inputs, input-oriented DEA is considered. Input oriented DEA assumes that DMUs have more power to control over the inputs than their service outputs. On the other hand, output-oriented DEA of technical efficiency is considered when the emphasis is on expanding outputs from a given level of inputs; it assumes DMUs can attract service users to their institutions through marketing, referrals, or by other means such as reputation on the quality of services [30]. In this study, output-oriented DEA was conducted to measure the technical efficiency of health centers in East Gojjam Zone. Output oriented DEA approach was preferred for these health institutions with less power of control to change their inputs than to their outputs; governmental resource allocation to such health intuitions is usually uniform since they are peer health DMUs.

There are two types of model assumptions designed to measure the technical efficiency of DMUs using DEA. The constant return to scale (CRS) (Charnes, Cooper, and Rhodes model) which assumes the scale of operation for a given DMU is not a factor for its productivity. The CRS model was then extended to a more flexible variable returns to scale (VRS) (Banker, Charnes, and Cooper model), which is appropriate when not all DMUs are assumed to operate at an optimal scale, considers the scale of each DMUs while computing their technical efficiency [1, 29].

The result from the analysis of VRS model takes two forms, decreasing returns to scale (DRS) and increasing returns to scale (IRS). DRS refer to a DMU is too large for the volume of activities it conducts. To run at the most productive scale size, a DMU exhibiting DRS must scale down its scale of operation. In contrast, a DMU with IRS is too small for its scale of operation. If a DMU is exhibiting IRS, it should expand its scale of operation to become an efficient DMU [32]. Since financial and regulatory constraints often result in a sub-optimal scale of operations, the VRS assumption of DEA was considered for this study to get a good estimation of efficiency among the health centers.

A DMU is said to be technically efficient when the point that represents the optimal mix of its inputs and outputs lies on the frontier line, when the technical efficiency score computed from the DEA equals 1. The health centers’ relative technical efficiency scores were obtained by solving the model given in Eq. 2. It is based on the output-oriented DEA with a VRS assumption which is described in [30, 33, 34].

$$\text{max}TEo\left(\text{U},\mathrm V\right)=\text{max}{\textstyle\sum_{i=1}^m}ViXio+V$$

Subject to

2 $$\begin{array}{c}\sum_{i=1}^mViXij-\sum_{r=1}^tUrYrj+\mathrm V\geq0;\mathrm j=1\dots\mathrm n,\\\sum_{r=1}^tUrYro=1,\\Ur,Vi\geq0\end{array}$$

where; Y_rj_ = the amount of output r produced by health facility j, X_ij_ = the amount of input i used by health facility j, U_r_ = the weight given to output r, (r = 1… t and t is the number of outputs), V_i_ = the weight given to input i, (i = 1… m and m is the number of inputs), j = the health facility under assessment.

### Identifying determinants of technical (in)efficiency

To see the effects of the organizational and environmental characteristics of the health centers on the technical (in)efficiency, the technical efficiency scores were transformed into inefficiency scores using Eq. 3 as in [35]. Then, we employed the tobit regression model (Eq. 4) since the dependent variable is censored at zero from below.

3 $$\mathrm{Inefficiency}\;\mathrm{score}=\left(\frac1{DEA\;score}\right)-1$$

The tobit regression model is expressed as:

$$Y_i=\left\{\begin{array}{c}Y_i^\ast=\alpha_\text{o}+\alpha_\text{i}X_\text{i}+\varepsilon_\text{i},\mathrm{if}\;Y_i^\ast>0\\0,\mathrm{if}\;Y_i^\ast\leq0;\mathrm i=1,2,3,\dots,\mathrm n;\varepsilon_\text{i}\sim\mathrm N(0,\delta^2)\end{array}\right.$$

For this study, the model can alternatively be stated as:

4 $$\mathrm{DEA}\;Ineff=\alpha_{\mathrm o}+\sum\nolimits_{i=0}^n\alpha\mathrm{iXi}+\varepsilon_\text{i}$$

where: ${Y}_{i}^{*}$ represents a possibly censored version of ${Y}_{i}$; $\alpha$_o_ represents a constant term; $\alpha$_i_ represents the vector of unknown regression parameters; X_i_ denotes the vector of independent variables; $\varepsilon$_i_ is the random error term; and DEA *Ineff* = (${Y}_{i}$) represents the technical inefficiency estimates of a health center.

## Results

### Organizational and environmental characteristics of the health centers

In this study, all selected health centers from the study area were assessed. Fourteen (52%) health centers have served for about 15 years and above and over 74% of health centers were serving a catchment population of more than 25,000. Majority of the health centers (70%) were in rural area and more than 80% of health centers were served by health mangers having BSC degree in health science fields (Table 1).

**Table 1:** Organizational and environmental characteristics of health centers in east Gojjam zone, Northwest Ethiopia, 2014 EFY (*N* = 27 DMUs)

No	Variable	Category	Frequency (N)	Percent (%)
1	Service year	≤ 15 years	13	48.15
		> 15 years	14	51.85
2	Catchment population	≤ 25,000	7	25.93
		> 25,000	20	74.07
3	Location of the health center	Rural	19	70.37
		Urban	8	26.63
4	Educational status of the head	Diploma	4	14.81
		Degree	22	81.48
		Masters	1	3.70
5	Managerial service year of the head	≤ 2 years	10	37.04
		> 2 years	17	62.96
6	Availability of functional health facility near to the health center with less than 2 km	Yes	25	92.59
		No	2	7.41
7	Average patient waiting time	≤ 20 min	5	18.52
		> 20 min	22	81.48

### Description of inputs and outputs

The 27 health centers provided 1,057,903 outpatient visits; 88,924 family planning visits; 10,327 referral (out) services; 12,768 skilled deliveries and 18,816 full vaccinations of children in the study year. These health services were produced by using 434 administrative staffs, 575 clinical staffs, 310 client beds and 895,429.9$ expense for recurrent materials including the pharmaceuticals (Table 2).

**Table 2:** Descriptive statistics for inputs and outputs of health centers in east Gojjam zone, Northwest Ethiopia, 2014 EFY (*N* = 27 DMUs)

Category	Variables	Sum	Mean	Std. Dev	Min	Max
Outputs	Number of outpatient visits	1,057,903	39,181.59	26,541.18	13,183	112,628
	Number of family planning visits	88,924	3293.48	1695.21	981	6615
	Number of referrals (referral out)	10,327	382.48	298.63	42	1303
	Number of skilled deliveries	12,768	472.89	296.24	140	1201
	Number of fully vaccinated children	18,816	696.89	392.53	3	1482
Inputs	Number of administrative staffs	434	16.07	3.02	11	25
	Number of clinical staffs	575	21.29	5.41	12	32
	Number of beds	310	11.48	5.24	2	26
	Expense for recurrent materials including pharmaceuticals in dollars	895,429.90	33,164.07	35,017.38	7662.49	200,059.10

### Efficiency scores

**Fig. 1:**
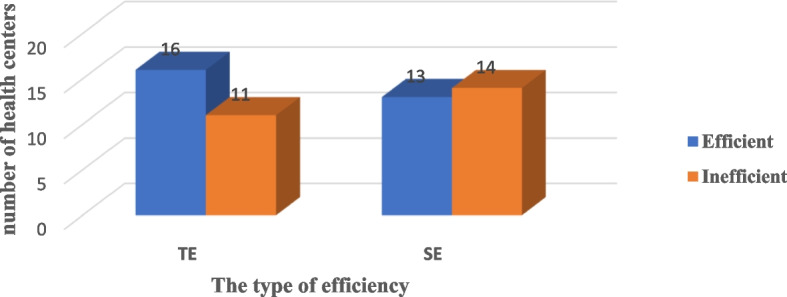
Technical and scale efficiency of health centers in east Gojjam zone, Northwest Ethiopia, 2014 EFY (*N* = 27 DMUs)

In this study, sixteen (59.3%) health centers were technically efficient, and thirteen (48%) health centers were scale efficient (Fig. 1)

The mean technical efficiency score of the health centers was found to be 0.899 ± 0.256 (Table 3).

**Table 3:** Efficiency scores of health centers in east Gojjam zone, Northwest Ethiopia, 2014 EFY (*N* = 27 DMUs)

Health center	Technical efficiency	Scale efficiency	Type of scale inefficiency
Fendiqa	1.00	1.00	-
Libanos	1.00	1.00	-
Giraram	0.86	0.87	IRS
Lega	1.00	0.39	IRS
Lumame	0.60	0.99	IRS
Wejel	0.53	0.99	DRS
Dejiba	0.57	0.96	IRS
Debre/elyas	0.78	0.99	DRS
Gofichima	1.00	0.71	IRS
Dejen	0.80	0.86	DRS
Yetnora	1.00	1.00	-
Hagere selam	0.91	0.92	DRS
Gindeweyin	1.00	1.00	-
Chemo	1.00	1.00	-
Geborie	1.00	1.00	-
Debre/qelemu	1.00	1.00	-
Amanuel	1.00	0.98	DRS
Gira/qidamin	0.90	0.71	IRS
Yejubie	1.00	1.00	-
Kork	0.59	0.99	DRS
Den	0.78	0.99	IRS
Weyira	1.00	1.00	-
Bichena	1.00	1.00	-
Yetmen	0.98	0.98	IRS
Debre/werq	1.00	1.00	-
Shiferie	1.00	1.00	-
Felege/birhan	1.00	1.00	-
Mean	0.89	0.94	
SD	0.16	0.14	
Min	0.53	0.39	
Max	1.00	1.00	

### Input reduction and output escalation projections to make relatively technical inefficient health centers to efficient

The average number of outpatient visits, family planning visits, referrals (referral out), skilled deliveries and fully vaccinated children required to make the relative technical inefficient health centers to be efficient were 22,433, 1,351, 155, 206 and 385, respectively. Similarly, the average number of administrative staffs, clinical staffs, beds, and the mean expense for recurrent materials including pharmaceuticals in dollars needed to make the relative technical inefficient health centers to efficient were 2, 3, 4 and 17,780$, respectively (Table 4).

**Table 4:** Input reduction and output escalation projections to make relatively technical inefficient health centers to efficient in east Gojjam zone, Northwest Ethiopia, 2014 EFY (*N* = 11 DMUs)

Category	Variables	Mean for the 11 inefficient health centers		
		Original	Projected	Difference
Outputs	Number of outpatient visits	35,386.82	57,819.82	22,433
	Number of family planning visits	2994.64	4345.82	1351
	Number of referrals (referral out)	365.64	520.27	155
	Number of skilled deliveries	352.18	557.73	206
	Number of fully vaccinated children	535.55	920.82	385
Inputs	Number of administrative staffs	16.82	14.91	-2
	Number of clinical staffs	21	19.36	-3
	Number of beds	12.73	8.72	-4
	Expense for recurrent materials including pharmaceuticals in dollars	45,297.02	27,517.27	-17,780

### Factors associated with the technical (in)efficiency of health centers

From the tobit regression model the catchment population, managerial service year, service year of the health center and number of motorcycles had negative association with the technical inefficiency of health centers whereas the number of administrative staffs and the number of clinical staffs had positive associations. The catchment population with β = -0.0000397, CI (-0.0000766, -2.87e-06) and number of administrative staffs with β = 0.1332697, CI = (0.0362345, 0.2303049) were found to be significantly associated with the technical inefficiency of health centers with 5% level of significance (Table 5).

**Table 5:** Factors associated with the technical inefficiency of health centers in east Gojjam zone, Northwest Ethiopia, 2014 EFY (*N* = 27 DMUs)

Tobits regression	Number of obs = 27					
	LR chi (6) = 17.20					
Log likelihood = -9.6217824	Prob > chi2 = 0.0086					
	Pseudo R2 = 0.4719					
Variables	Coefficient	Std. Err	T	*P* > / t /	95% Conf. Interval	
Service year	-0.03	0.02	-1.99	0.06	-0.06	0.01
Catchment population	-3.97e-05	1.77e-05	-2.24	0.04*	-7.66e-05	-2.87e-06
Managerial service year	-0.04	0.04	-0.94	0.36	-0.13	0.05
Number of admin staffs	0.13	0.05	2.86	0.01*	0.04	0.23
Number of clinical staffs	0.04	0.03	1.53	0.14	-0.01	0.09
Number of motorcycles	-0.13	0.14	-0.91	0.37	-0.43	0.17
Constant	-1.02	0.63	-1.62	0.12	-2.34	0.29
Sigma	0.34	0.07			0.18	0.50

^*^Significant with 5% level of significance

## Discussion

Performance measurement seeks to monitor, evaluate, and communicate the extent to which various aspects of the health system meet their key objectives. Its role is to improve the quality of decisions made by all actors within the health system. Generally, the common health performance measures include; population health, individual health outcomes, quality and appropriateness of care, responsiveness of health system, equity, and productivity/efficiency [36]. This study assessed the technical efficiency of health centers which is one of the performance measures in the health care delivery system. Technical efficiency was computed using health centers inputs (number of administrative staffs, number of clinical staffs, number of beds and expense for recurrent materials including pharmaceuticals) and outputs (number of outpatient visits, number of family planning visits, number of skilled deliveries, number of referrals and number of fully vaccinated children).

Overall, the mean technical efficiency of the health centers was 0.899 with a standard deviation of 0.156. This finding is consistent with the study conducted on public hospitals in Northwest Ethiopia [18] with the mean technical efficiency score of 0.92. However, it is lower than the study on public hospitals in Eritrea [34] with mean technical efficiency score of 0.97, and higher than studies on health centers in Southwest Ethiopia [19] and health posts in Tigray region [16] with the mean technical efficiency score of 0.76 and 0.57 respectively. it is also higher than studies conducted on health centers in Gambia [37], hospitals in China [38], hospitals before the implementation of the health sector evolution plan in Iran [39] with the mean technical efficiency scores of 0.65, 0.79, and 0.86 respectively. This might be due to the use of a lower number of DMUs in our study, resulting in higher efficiency scores of health centers relative to the earlier studies [40]. This difference might be also due to the variation in the level of health facilities and the health care systems across areas.

Fourteen (52%) health centers were scale inefficient and among the scale inefficient health centers, 8 (57%) were in increasing return to scale. This implies that these eight health centers were below the optimal scale of operation, and they need to scale up to become efficient as their peer efficient health centers.

In this study, the average number of outpatient visits, family planning visits, referrals, skilled deliveries, and fully vaccinated children required to make the relative technical inefficient health centers to be efficient were 22,433, 1,351, 155, 206 and 385, respectively. This indicates that these inefficient health centers could produce more 22, 433 outpatient visits, 1,351 family planning visits, 155 referrals, 206 skilled deliveries and 385 fully vaccinations of children.

From the tobit regression analysis, a 10,000 increase in catchment population increases the technical efficiency of health centers by 0.397. This finding is consistent with the study on health centers in Southwest Ethiopia [19] where a 10,000 increase in catchment population increases the technical efficiency of health centers by 0.2. However, it is contrary to the study on public hospitals in Northwest Ethiopia [18] in which a 100,000 increase in catchment population decreases the TE of hospitals by 0.0524. This may be due to higher number of health service users from the broad catchment population which results in more service outputs, and this perhaps increase the technical efficiency of health centers in our study. A unit increase in the number of administrative staffs decreases the technical efficiency of health centers by 0.13. This may be due to the hiring of too many admirative employees and underuse of these staffs which can result in lower efficiency of health centers.

This study has some limitations. First the DEA is a deterministic approach in which the efficiency of a DMU is assumed to be estimated by the ratio of its outputs and inputs. However, there are indeterministic factors which may influence the technical efficiency of health centers like civil war or natural disasters including epidemics. Besides this, we used the 2022 data for the efficiency analysis and the result cannot be applied for the current decision making though it shows the performances of the health centers for the 2022 year. We also used the self-reported data of health centers which may bias the estimation of the efficiency scores. Despite these limitations, this study can be used as a baseline for further studies in the area.

## Conclusions

The mean technical efficiency of health centers in East Gojjam zone, Northwest Ethiopia was very high. However, nearly half of the health centers were technically inefficient, which indicates the exitance of a space for further improvements in the productivity of these health centers. Inefficient health centers could provide more 22, 433 outpatient visits, 1,351 family planning visits, 155 referrals, 206 skilled deliveries and 385 fully vaccinations of children if they were efficient as their peer efficient health centers. It is better to employ optimal number of administrative staffs and constructing health centers on appropriate sites to have high catchment population coverage to improve the productivity of the health centers. Here, sites where health centers be constructed should give due attention by the responsible bodies so that the standard requirement of population coverage for health centers [41] (i.e. 25,000 in rural and 40,000 in urban) need to be fulfilled. In doing this, we can eliminate wastage of resources on the one hand and unmet need of health services on the other hand if health facilities provide health services according to their expectations.

### Supplementary Information

**Supplementary Material 1.**

**Supplementary Material 2.**

## Data Availability

All data analysed during this study are included in the supplementary file (Additional file 2).

## Abbreviations

CRS: Constant return to scale
DEA: Data envelopment analysis
DMUs: Decision making units
DRS: Decreasing return to scale
ETB: Ethiopian birr
IRS: Increasing return to scale
SFA: Stochastic frontier analysis
TE: Technical efficiency
US$: United states dollar
VRT: Variable return to scale
