# Supplementary material for: Level of technical efficiency and associated factors among health centers in East Gojjam Zone, Northwest Ethiopia: an application of the data envelopment analysis
Source: BMC Health Serv Res. 2024 Mar 21;24:361. doi: 10.1186/s12913-024-10843-1 (PMC10956267; doi:10.1186/s12913-024-10843-1)
Supplement: Supplementary file 1 — Supplementary Material 1. [file 12913_2024_10843_MOESM1_ESM.docx]

## Additional file 1: the questionnaire used for the study on level of technical efficiency and associated factors among health centers in East Gojjam Zone, Northwest Ethiopia

## QUESTIONNAIRE

DEBRE MARKOS UNIVERSITY
COLLEGE OF MEDICINE AND HEALTH SCIENCES
DEPARTMENT OF PUBLIC HEALTH

Good morning/ Good afternoon!

I am ____________ who came from _______________; I am a data collector of the research
Project entitled “Technical Efficiency and associated factors of health centers in East Gojjam Zone, Northwest Ethiopia”. I expect that you will freely give me whatever information available in your health center according to the checklist. The information that you give me will be very useful for this study. This information will help policy makers to design how to allocate resources and measure the performance of the health centers based on the research findings.

Are you voluntary participating in the study on behalf of your health centers?

1. Yes
2. No

**Thank you for your voluntary participation!!!**

**Section I: General information about the health center**

| Questions | Response | Remark |
| --- | --- | --- |
| The health center’s code/ID/name |  |  |
| How many years since establishment? (years of service) |  |  |
| Total catchment population of the health center |  |  |
| Is there a health facility near to your health center? | 1. Yes  2. No |  |
| If yes for Q 105 in what distance in KM? |  |  |
| If yes for Q 105 What type? | 1. Primary Hospital  2. General Hospital  3. Specialized Hospital  4. private clinic |  |
| Location of the health center | 1. Rural 2. Urban |  |
| What is the maximum qualification (educational status) of the head of the health center? |  |  |
| How many years the head of the health center served as a manager? |  |  |
| The average outpatient waiting time for health services in the health centers? |  |  |

**Section II: Inputs used by the health center**

1. **Recurrent expenses**
2. Administrative and maintenance

| Reason for expense | Total expense in birr in the 2014 Ethiopian fiscal year |
| --- | --- |
| Stationary |  |
| Electricity |  |
| Telephone service |  |
| Water |  |
| Building maintenance |  |
| Gasoline and Lubricants |  |
| Total |  |

1. Drugs and medical supplies

| Reason for expense | Total expense in birr in the 2014 Ethiopian fiscal year |
| --- | --- |
| Drugs |  |
| Vaccines |  |
| Medical supplies |  |
| Chemical & regents |  |
| Total |  |

1. **Labour**

| Human resource | Quantity in the 2014 Ethiopian fiscal year |
| --- | --- |
| Administrative staff |  |
| Clinical staff  Nurse  Health officer  Physician  Midwifes  Lab technician/technologist  Pharmacist |  |
| Total |  |

1. **Capital**

| Capital items | Quantity in the 2014 Ethiopian fiscal year |
| --- | --- |
| Beds in the health center |  |
| Vehicles;  Ambulances  Motorcycles |  |
| Total |  |

**Section III: Outputs in the health center**

| Type of output | Quantity in the 2014 Ethiopian fiscal year |
| --- | --- |
| Total number patients visited the outpatient department |  |
| Total number of clients visited the family planning department |  |
| Total number of referrals (referral out) |  |
| Total number of deliveries |  |
| Total number of fully vaccinated children |  |
